# Influence of Extraction Method on the Bioactivity of *Pistacia vera* L. Extracts: Metabolic and Gene Expression Changes in Colorectal Cancer Cells

**DOI:** 10.3390/foods15020205

**Published:** 2026-01-07

**Authors:** Giulia Zerbo, Paolo Giuseppe Bonacci, Morena Terrana, Valentina Greco, Gianfranco Cavallaro, Carmela Bonaccorso, Alessandro Giuffrida, Cosimo Gianluca Fortuna, Nicolò Musso

**Affiliations:** 1 Department of Chemical Sciences, Università degli Studi di Catania, Viale Andrea Doria, 95123 Catania, Italy; giulia.zerbo@unict.it (G.Z.); valentina.greco@unict.it (V.G.); gianfranco.cavallaro@phd.unict.it (G.C.); carmela.bonaccorso@unict.it (C.B.); alessandro.giuffrida@unict.it (A.G.); cg.fortuna@unict.it (C.G.F.); 2 Department of Biomedical and Biotechnological Sciences, Università degli Studi di Catania, Via Santa Sofia, 95123 Catania, Italy; morena.terrana@ior.it; 3 Advanced and Innovative Diagnostic Academy (A.I.D.A.), Via Santa Sofia, 95123 Catania, Italy; nicor1@icloud.com; 4 Department of Medicine and Surgery, Università Kore di Enna, Contrada Santa Panasia, 94100 Enna, Italy

**Keywords:** *Pistacia vera* L., colorectal cancer, green extraction methods, glucose metabolism, inflammatory pathway, HCT-116 cells

## Abstract

Sustainable extraction methods represent a key strategy in green chemistry and nutraceutical development, aiming to replace conventional solvent-based techniques while maintaining extract quality and safety. This study compared pistachio (*Pistacia vera* L.) extracts obtained by Ultrasound-Assisted Extraction (UAE) and a classical solvent-based protocol, focusing on compositional features and biological effects. Extracts were characterized for their chemical profiles, and their impact on HCT-116 colon-derived cells was evaluated through viability assays and gene expression analysis. The UAE-derived extract, richer in carbohydrates, promoted higher cell proliferation after 72 h, whereas the classical extract upregulated *HMOX-1*, suggesting activation of antioxidant defense pathways. Moreover, UAE treatment downregulated *GLUT2* expression while modulating cytokinestranscripts, indicating a possible carbohydrate-driven immunometabolic response. Overall, these findings highlight both the advantages and limitations of green extraction approaches: while environmentally sustainable and efficient, ultrasound-assisted protocols may modify extract composition in ways that influence biological responses. Optimization of extraction parameters is therefore essential to ensure a balance between ecological sustainability, compositional integrity, and biological safety.

## 1. Introduction

In recent decades, interest in plant-derived compounds with health-promoting properties has increased markedly, particularly those obtained from edible species with established nutritional value. Pistachio (*Pistacia vera* L.) has gained attention for its nutritional qualities and emerging nutraceutical potential. Its cultivation holds considerable economic relevance and generates edible nuts as well as substantial lignocellulosic by-products (e.g., hulls, leaves, shells), which are increasingly explored for biotechnological applications [1]. Pistachio seeds contain phenolic acids, flavonoids, stilbenes, terpenoids, and tocopherols—bioactive compounds associated with antioxidant, anti-inflammatory, antimicrobial, vasoprotective, and cardioprotective effects [2], supporting their inclusion in the Mediterranean diet and functional food formulations [3]. Within this framework, the objective of the present study is to investigate how different extraction methodologies—both conventional and green—influence the chemical composition, antioxidant properties, and cellular responses of pistachio-derived extracts. Clarifying these effects is essential for developing sustainable and well-characterized nutraceutical preparations suitable for biomedical applications. Traditional extraction techniques such as maceration, Soxhlet extraction, and reflux extraction require substantial solvent volumes (e.g., n-hexane, acidified methanol) and energy input. These limitations have driven a shift toward greener technologies, including ultrasound-assisted, microwave-assisted and critical superfluid extraction —which reduce solvent consumption, processing time, and environmental impact while improving phytochemical preservation [4]. In this context, the European Green Deal has received considerable attention [5,6] for promoting chemical practices aligned with environmental protection and sustainability. The transition from conventional methods to greener alternatives contributes to reducing toxic solvent use while facilitating the valorization of agro-industrial residues [7]. Despite technological advances, extracts intended for nutraceutical or biomedical use require rigorous compositional characterization. This aspect is particularly critical in oncology, where insufficient extraction selectivity may result in the unintended co-extraction of compounds capable of modulating metabolic pathways, ultimately affecting cellular responses [8,9]. The relevance of phytochemicals in cancer research is well established: several plant-derived molecules, such as Vinca alkaloids from Catharanthus roseus [10,11] and camptothecin derivatives [12], are already used as chemotherapeutic agents, while others—including curcumin [13] and resveratrol [14]—are currently under investigation as adjuvant compounds. Within this scientific context, comparing extraction strategies becomes essential to elucidate how methodological choices influence the biochemical profile and biological activity of pistachio-derived extracts. Such knowledge is fundamental for the development of well-defined, sustainable preparations with potential applications in disease-modifying interventions.

## 2. Materials and Methods

### 2.1. Pistachio Preparation and Extraction

Pistachio sample was obtained from Bronte (Sicily, Italy). Prior to polyphenolic extraction, all samples underwent a standardized defatting procedure to remove lipophilic components that could interfere with subsequent extraction and analysis. Following established protocols [15] finely chopped pistachio samples (5 g) were subjected to lipid extraction using n-hexane (10 mL) under continuous agitation for 24 h at room temperature. The mixture was subsequently allowed to settle by gravity separation for 2–3 h, after which the supernatant containing dissolved lipids was carefully decanted and discarded. The remaining defatted pistachio material was filtered under vacuum using a Buchner funnel equipped with filter paper to remove residual n-hexane and achieve complete solvent elimination. This defatting step served as a prerequisite for both extraction methodologies employed in this study, ensuring that subsequent polyphenolic extraction procedures would target the desired hydrophilic and amphiphilic bioactive compounds without interference from the substantial lipid fraction naturally present in pistachio kernels.

First Method (Green Method). According to the literature [16] the pistachio sample previously defatted (1 g, weighed on an analytical digital balance), was extracted using a 45% *v*/*v* ethanol/water solution by means of Ultrasound-Assisted Extraction (UAE). The extraction was carried out in a heated ultrasonic bath (LBX-ULTR series, Labbox, Spain; 40 kHz frequency, 3 L capacity, 120 W ultrasonic power) maintained at 50 °C for 15 min. After extraction, the sample was centrifuged at 5000 rpm for 30 min at 5 °C to remove residual fats and coarse particulates. The recovered supernatant was then concentrated using a rotary evaporator, and the resulting fraction was stored in a freezer. Ultimately, 88 mg of extract was obtained from the first green extraction method.

Second Method (Classic Method). According to the literature [15] the previously defatted pistachio sample (5 g, accurately weighed on an analytical digital balance) was mixed with 50 mL of methanol containing 0.1% (*v*/*v*) HCl. The mixture was sonicated for 15 min and subsequently centrifuged (5000 rpm, 10 min, 4 °C). The resulting pellet was extracted twice more under the same conditions. The methanolic fraction was pooled and concentrated using a rotary evaporator. The residues were then dissolved in 20 mL of distilled water and extracted four times with 20 mL of ethyl acetate in a separating funnel. The organic phase obtained was collected and dried with Na_2_SO_4_ for 20 min. Ultimately, 90 mg of extract was obtained from the second classic method.

At the end of the extraction protocol, the dry extract obtained from both the green and classical methods was resuspended in methanol to allow the preparation of standardized aliquots with a fixed and known mass of extract. Specifically, each microvial was loaded with exactly 1 mg of extract, solubilized in methanol to allow precise volumetric handling. Following aliquoting, the methanol was completely removed by evaporation, yielding a dry, pre-weighed extract ready for resuspension prior to cell treatment.

$$\mathrm{Extraction}\;\mathrm{Yield}\;(\%)=\frac{Mass\;of\;dry\;extract\;\left(g\right)}{Initial\;mass\;of\;sample\;\left(g\right)}\times 100$$

### 2.2. Total Carbohydrate Quantification

Total carbohydrate content of pistachio extracts was determined using the phenol-sulfuric acid colorimetric method, described by DuBois et al. [17] and adapted by Nielsen [18]. A standard curve was prepared using D-glucose as reference. Briefly, a glucose stock solution (100 mg/mL) was prepared by dissolving 500 mg of D-glucose in 5 mL of distilled water. Standard solutions ranging from 80 to 800 µg/mL were prepared by diluting appropriate volumes (10, 25, 50, 75, and 100 µL) of the stock solution, each combined with 100 µL of 5% (*w*/*v*) phenol solution and 500 µL of concentrated sulfuric acid (96–98%), and brought to a final volume of 5 mL with distilled water. After a 10 min incubation at room temperature, absorbance was measured at 490 nm using a spectrophotometer.

For sample analysis, pistachio extracts were dissolved in methanol to achieve a final concentration of 20 mg/mL. Aliquots of 100 µL were subjected to the same colorimetric procedure: samples were mixed with 100 µL of 5% phenol solution and 500 µL of concentrated sulfuric acid, incubated for 10 min at room temperature, then diluted to a final volume of 2 mL with distilled water. Absorbance was measured at 490 nm. Total carbohydrate content was calculated from the glucose standard curve (y = 0.0013x + 0.0483; R^2^ = 0.9943) and expressed as glucose equivalents per 100 mg of dry extract (% *w*/*w*). All measurements were performed in triplicate.

### 2.3. Polyphenolic Composition Analysis by HPLC-MS

The phenolic profile of pistachio extracts was characterized using high-performance liquid chromatography coupled with mass spectrometry (HPLC-MS) to identify the major bioactive compounds and assess method-dependent compositional differences. All mass spectrometry experiments were performed using an LTQ XL mass spectrometer equipped with an H-ESI II source (ThermoFisher, San Jose, CA, USA) operating in positive ionization mode. The spray voltage was set to 3 kV, capillary temperature to 250 °C, capillary voltage to 20 V, and tube lens to 120 V. Full scan mode was employed over the *m*/*z* range 150–2000.

Chromatographic separation was performed on a Thermo Scientific Vanquish HPLC system (Thermo Scientific, Bremen, Germany) equipped with a Phenomenex Kinetex C-18 column (100 mm × 2.10 mm, 2.6 μm). The mobile phases consisted of water containing 0.1% formic acid (A) and acetonitrile containing 0.1% formic acid (B). The gradient elution program was as follows: 0 min, 10% B; 60 min, 100% B (held for 10 min). The flow rate was 0.25 mL/min, injection volume was 10 μL, and column temperature was maintained at 40 °C, according to previously established methods with minor modifications [19]. For sample analysis, lyophilized pistachio extracts were dissolved in methanol to obtain a stock solution of 2 mg/mL. For HPLC-MS analysis, 10 μL of stock solution were diluted to 1 mL with methanol, resulting in a final concentration of 20 μg/mL. Samples were filtered through 0.22 μm nylon syringe filters prior to injection.

### 2.4. Cell Culture and Treatments

A human colorectal cancer cell line (HCT-116, Cat. No. CCL-247, ATCC^®^, Manassas, VA, USA) was cultured in McCoy’s 5A (Cat. No. MS029E1001, Biowest, Nuaillé, France) medium supplemented with 10% of fetal bovine serum (FBS, Cat. No. S1560-500, Biowest, Nuaillé, France) and penicillin/streptomycin (50 μg/mL, Cat. No. L0022-100, Biowest, Nuaillé, France) at 37 °C in a controlled atmosphere with 5% CO_2_ according to the supplier’s instructions.

### 2.5. Impact of Extracts on Cell Viability and Proliferation

Both pistachio extracts were solubilized in a 1:1 (*v*/*v*) mixture of PBS (Cat. No. 10010023, Gibco™, Thermo Fisher Scientific, Waltham, MA, USA) and 70% ethanol, and tested for their metabolic impact at a final concentration of 250 ng/μL [20,21]. Treatments were performed the day after seeding cells by adding 10 μL of extract solution directly into 96-well plates resulting in a final concentration of 250 ng/µL.

HCT-116 cells counted with Bio-Rad TC20 Automated Cell Counter (Cat. No. 1450102, BioRad, Hercules, CA, USA, 94547-1811) were seeded at a density of 5000 cells per well in McCoy’s 5A medium supplemented with 10% FBS, 1% Penicillin-Streptomycin, according to ATCC recommendations. Cells were treated with the green extract or the classic extract for 24, 48, and 72 h. Untreated cells were included in each assay and used as controls.

Cell viability was assessed using the MTT assay [22]. At each time point, 10 μL of MTT solution (5 mg/mL in 1× PBS), prepared from powder (Cat. No. M5655, Sigma-Aldrich, St. Louis, MO, USA), was added to each well and incubated for 3 h at 37 °C in a humidified incubator with 5% CO_2_. Then, supernatant was removed and formazan crystals were solubilized with dimethyl sulfoxide (DMSO, Sigma-Aldrich), and absorbance was measured at 570 nm using a BioTek Synergy H1 Multimode Reader (Agilent Technologies, Santa Clara, CA, USA). All experimental conditions and time points were performed in quadruplicate (technical replicates) and repeated in at least three independent biological experiments.

### 2.6. Impact on Inflammatory and Metabolic Pathways

To evaluate extract impact on Inflammatory and Metabolic pathway, HCT-116 cells were seeded in 6-well plates at a density of 200,000 cells per well and incubated overnight under standard culture conditions. The following day, cells were treated for 24 h with either the green extract or the classic extract at a final concentration of 250 ng/μL. In addition to the two pistachio-derived extracts, a heteroarylethylene compound named PB4, previously synthesized and characterized in our laboratory [22,23,24], was included for comparative purposes. PB4 has been reported in our earlier publications to possess significant cytotoxic activity (IC_50_ = 0.32 μM [22]) and to markedly influence cellular oxidative and inflammatory processes [25]. In this work, it was tested at a concentration equal to half its IC_50_ for 24 h to simulate an inflammatory but non-lethal environment for the cells. After treatment, cell culture supernatants were removed, and cells were lysed directly in the wells by adding RLT buffer supplemented with β-mercaptoethanol, following the manufacturer’s instructions for the QIAGEN RNeasy Mini Kit (Ref. 74104, QIAGEN, 40724 Hilden, Germany). The lysates were processed immediately for total RNA extraction to allow subsequent analysis of genes involved in different pathways. All experimental conditions were tested in three independent biological experiments.

### 2.7. RNA Extraction and cDNA Synthesis

RNA extraction was carried out following the manufacturer’s instructions provided by QIAGEN RNeasy Mini Kit (Ref. 74104, QIAGEN, 40724 Hilden, Germany). RNA was quantified using both the Eppendorf BioPhotometer^®^ D30 and the fluorimeter Qubit RNA HS Assay Kit to evaluate purity and quantity of the initial sample, respectively (Ref. 32850, Invitrogen, 92008 Carlsbad, CA, USA). The samples with an A260/A280 ratio between 1.8 and 2.1 were considered suitable for downstream applications. Further RNA quality analysis was performed by evaluating the RNA Integrity Number (RIN) of RNA samples extracted with the Agilent RNA 6000 Nano Kit (Cat. No. 5067-1511, Agilent, Santa Clara, CA 95051, United States) on the Agilent Bioanalyzer 2100 platform (Table S3). For cDNA synthesis, 1 μg of total RNA was reverse transcribed using the QuantiTect Reverse Transcription Kit (Qiagen, Cat. No. 205311). This kit includes an integrated genomic DNA wipeout step to eliminate potential DNA contamination and a mix of oligo-dT and random primers to ensure efficient and unbiased cDNA synthesis. The resulting cDNA was stored at –20 °C.

### 2.8. RT-qPCR

Gene expression measurements were performed by means of mRNA quantification using a LightCycler^®^ 480 II instrument (Roche, Switzerland) using QuantiTect SYBR Green PCR Kit (Cat. No. 204143 QIAGEN, Germany) and cDNA per well (50 ng total per well). Real-time cycler conditions: Initial Activation Step, 15 min, 95 °C. 3-step cycling × 40 cycles: Denaturation, 15 s, 94 °C; Annealing, 30 s, 55 °C; Extension, 30 s, 72 °C (Fluorescence Acquisition in this step). As a negative control, a reaction in the absence of cDNA (no template control, NTC) was performed. All primers in this studio are QuantiNova LNA PCR Assay (Table 1, Cat. No. 249990, QIAGEN GmbH, Gilden, Germany). Target gene expression levels (*GLUT2*, *IL-6*, *IL-10*, *HMOX-1*) were normalized to the housekeeping gene RPLP0, and relative quantification was performed using the ΔΔCt method. Data analysis was carried out using the LightCycler^®^ 480 software (version 1.5). The relative RNA expression level for each sample was calculated using the well-known 2^−∆∆CT^ method in which the threshold cycle (CT) value of the gene of interest is compared to the CT value of our selected internal control (RPLP0) [26]. RT-PCR amplifications were performed in three technical replicates [27].

## 3. Results

### 3.1. Comparative Chemical Results Between the Extracts

Given that both extraction protocols utilize solvent systems with well-established affinity for bioactive polyphenolic compounds, we focused our comparative analysis on extraction efficiency as the primary discriminating factor for preliminary method assessment. The extraction yield for the first method (green method) is about 8.8%. The extraction yield for the second method (classic method) is about 1.8% (Table 2). Based on the extraction yields, the UAE with ethanol/water (45% *v*/*v*) proved to be more efficient. In contrast, the second approach, consisting of an initial extraction with acidified methanol followed by liquid–liquid partitioning with ethyl acetate, was more complex and time-consuming. Although this method introduces additional purification steps (centrifugation, ethyl acetate partitioning, and drying over sodium sulfate) which may enhance selectivity by removing unwanted components and improving the purity of the final product, it resulted in lower overall yields.

### 3.2. Semi-Quantitative Polyphenolic Profile

The extraction step represents a critical phase in phytochemical analysis, as the choice of extraction methodology and conditions profoundly influences the composition of the final extract and the recovery of target bioactive compounds [28,29]. For comparative evaluation of the two extraction methods, four major phenolic compounds, consistently present as the most abundant peaks across the two extracts (green and classic), were selected for identification and semi-quantitative comparison. These compounds were identified based on retention time, mass, and comparison with literature data on *Pistacia vera* L. phenolic composition. Chlorogenic acid was unambiguously confirmed by co-elution with an authentic standard (retention time 61.3 min, *m*/*z* 397 corresponding to [M+H+CH_3_CN]^+^). The remaining compounds were assigned as follows: rutin (quercetin-3-O-rutinoside, RT 58.3 min, *m*/*z* 680 [M+Na+HCOOH]^+^), a luteolin derivative (RT 45.5 min, *m*/*z* 527), and procyanidin trimer (RT 67.3 min, *m*/*z* 850 [M+H−H_2_O]^+^).

Semi-quantitative analysis was performed by calculating relative peak area percentages, assuming similar ionization efficiency for structurally related phenolic compounds. HPLC-MS chromatograms for the two extracts are provided in supplementary figures (Figures S1 and S2).

Results are expressed as percentage of total phenolic content (Table 2). Rutin was the predominant phenolic compound in both extracts, accounting for more than 60% of total phenolic content, followed by chlorogenic acid (5.8% in classic vs. 16.2% in green extract) and minor components. Notably, method-dependent selectivity was observed: the classic extraction method enriched procyanidin trimer in pistachio extracts (14.1% in classic vs. 6.1% in green extract), while chlorogenic acid content was significantly reduced (5.8% in classic vs. 16.2% in green extract), suggesting selective loss during liquid–liquid partitioning or preferential extraction of oligomeric phenolics.

### 3.3. Carbohydrate Enrichment in Pistachio Extracts

Quantitative analysis revealed substantial carbohydrate content in the obtained extracts, with values significantly higher than those typically reported for whole pistachio kernels in the literature. Specifically, the Bronte green extract showed the highest carbohydrate content at 33.6% (expressed as glucose equivalents), followed by the Bronte classical extract at 25.5% (Table 3). It is important to note that these values represent the carbohydrate content within the obtained extracts themselves, rather than the total carbohydrate content of the original pistachio kernels. This apparent enrichment is a direct consequence of the extraction methodology employed. Both extraction protocols selectively co-extract water-soluble and polar compounds, including mono-, oligo-, and polysaccharides, alongside the target phenolic compounds. The UAE method, utilizing aqueous ethanol (45% *v*/*v*), exhibits particularly strong affinity for hydrophilic carbohydrates, resulting in their preferential concentration in the final extract relative to less polar constituents that remain in the defatted matrix.

When these extract-based percentages are normalized to the original pistachio mass using extraction yields (Table 3), the calculated whole-kernel carbohydrate recoveries become more consistent with literature values for *Pistacia vera* L. However, the key finding remains that green extraction methods yield extracts with substantially higher carbohydrate-to-polyphenol ratios compared to classical protocols, independent of absolute recovery.

### 3.4. Metabolic Impact of Green and Classic Extracts

**Figure 1:**
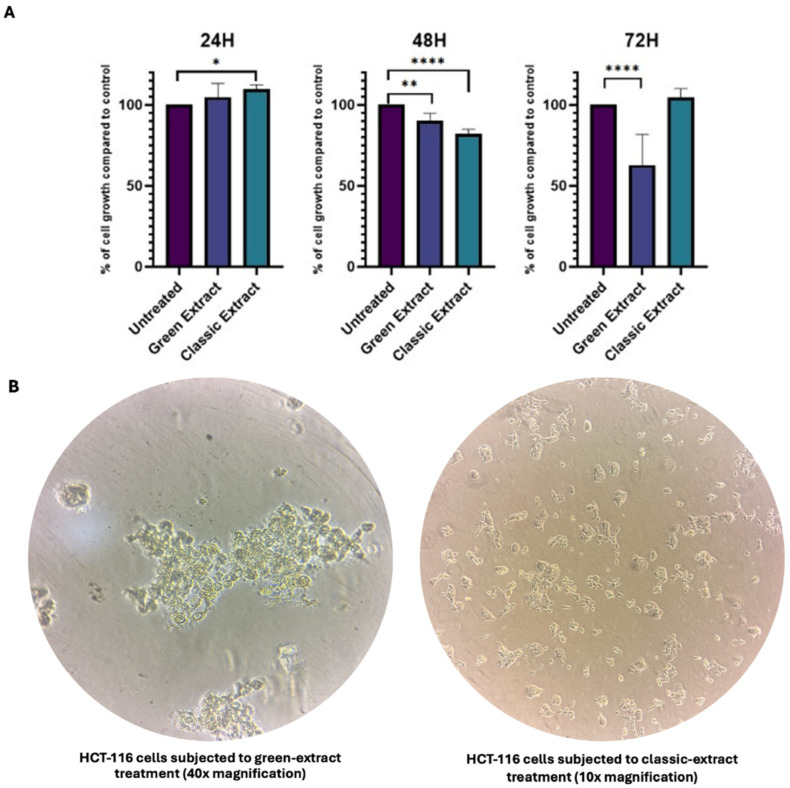
(**A**) Effect of the green extract and the classic extract extracts on HCT-116 cell viability after 24, 48, and 72 h of treatment. Cells were treated with the green extract (indigo bars) or the classic extract (blue bars) at a final concentration of 250 ng/μL. Untreated cells (purple bars) were used as controls. Cell viability was assessed via MTT assay and results are expressed as percentage relative to control (set to 100%). Data are presented as mean ± Standard Deviation (SD) (*n* = 4). Statistical significance was determined by Ordinary one-way ANOVA multiple comparisons test: * *p* < 0.05; ** *p* < 0.01; **** *p* < 0.0001. (**B**) Representative phase-contrast images of HCT-116 cells after 72 h of treatment. Cells exposed to the green extract ((**left**), 40× magnification) exhibited marked hyperproliferation followed by detachment from the culture surface, whereas those treated with the classic extract ((**right**), 10× magnification) maintained a more adherent phenotype with lower overall cell density.

To evaluate the metabolic impact of both pistachio extracts on HCT-116 cells, an MTT assay was conducted after 24, 48, and 72 h of treatment at a final concentration of 250 ng/μL (Figure 1, Table S1). At 24 h, no significant difference in cell viability was observed between the green extract-treated and control (untreated) cells (average growth 104.8%, *p*-value > 0.05). After 48 h, the green extract induced a modest but statistically significant reduction in cell viability, with treated cells displaying approximately 96% viability compared to control (*p* = 0.0141). At 72 h, a marked decrease in viability was detected, with treated cells showing only 62.5% viability relative to control (*p* < 0.0001). However, this result was not associated with cytotoxicity. Bright-field microscopy revealed extensive cellular overgrowth and detachment in treated wells at 72 h (Figure 1B), suggesting that the reduced MTT signal was due to hyperproliferation-induced confluence and subsequent detachment, rather than direct cytotoxic effects of the extract.

As for the classic extract, after 24 h treated cells exhibited a slight but statistically significant increase in metabolic activity compared to untreated controls, reaching 109.65% viability (*p* = 0.0226). At 48 h, a significant reduction in cell viability was observed, with treated cells showing 82.13% viability relative to control (*p* < 0.0001). However, at 72 h, cell viability returned to levels comparable to untreated cells (104.69%), with no statistically significant difference (*p* > 0.05). These findings indicate a transient inhibitory effect at 48 h, followed by a recovery of metabolic activity over time.

### 3.5. Impact on Inflammation Pathway

**Figure 2:**
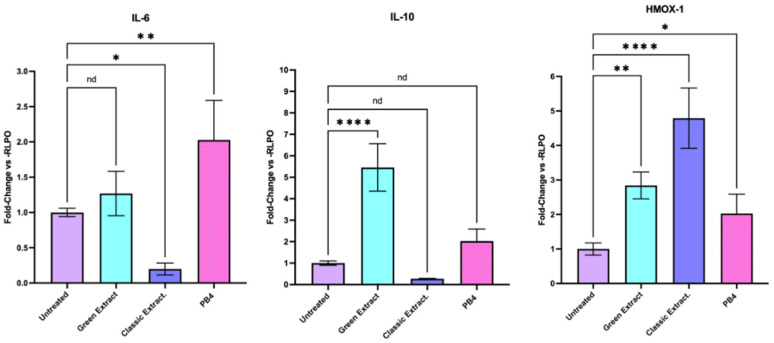
Effects of pistachio-derived extracts on the expression of *IL-6* (Interleukine 6), *IL-10*, (Interleukine 10) and *HMOX-1* (Heme—Oxygenase 1) genes in HCT-116 cells after 24 h of treatment. Gene expression levels were assessed by RT-qPCR and normalized to the housekeeping gene RPLP0. Results are expressed as fold change relative to untreated control cells (CTRL). Cells were treated with two types of extracts: the one extracted using a green extraction method, and the one extracted using conventional extraction, and PB4. Statistical significance was determined by Two-stage linear step-up procedure of Benjamini, Krieger and Yekutieli: * *p* < 0.05; ** *p* < 0.01; ; **** *p* < 0.0001; nd: not detectable.

The expression levels of *HMOX-1*, *IL-6*, and *IL-10* were assessed by RT-qPCR in HCT-116 cells treated for 24 h with the green extract or the classic extract at a final concentration of 250 ng/μL. Gene expression was normalized to the housekeeping gene *RPLP0* and reported as fold change relative to untreated control cells (Figure 2, Table S2). Regarding cytokines, the green extract treatment induced a significant increase in *IL-10* expression (5.04) relative to control, demonstrating its antioxidant activity mediated by chlorogenic acid [30,31,32]. The classic extract, on the other side, significantly downregulated the expression of *IL-6* (0.19) or *IL-10* (0.27), with the suppression of *IL-6* likely mediated by the enrichment in procyanidin trimers characteristic of the classical extraction method [33,34]. Treatment with the classic extract resulted in a marked upregulation of *HMOX-1* expression, with a relative fold change of 4.85 compared to control. In contrast, *HMOX-1* expression in the green extract-treated cells was 2.90. The differential phenolic profiles correlate with observed biological responses. The classic extract, characterized by enrichment in procyanidin oligomers, exhibited significantly enhanced antioxidant activity mainly mediated by *HMOX-1* upregulation. This superior bioactivity reflects not higher total phenolic content—which was comparable between methods—but rather selective enrichment of bioactive oligomeric phenolics known for strong Nrf2-activating properties [34]. These results suggest that the green and classic extracts exert distinct effects on gene expression, with the classic extract preferentially inducing an antioxidant response through *HMOX-1* upregulation and *IL-6* suppression, while the green extract mainly promoting immunomodulatory cytokine expression (*IL-10*), underscoring that extraction selectivity, rather than total phenolic yield, determines the biological efficacy profile of pistachio-derived nutraceuticals.

### 3.6. Impact on Glucose Pathway

**Figure 3:**
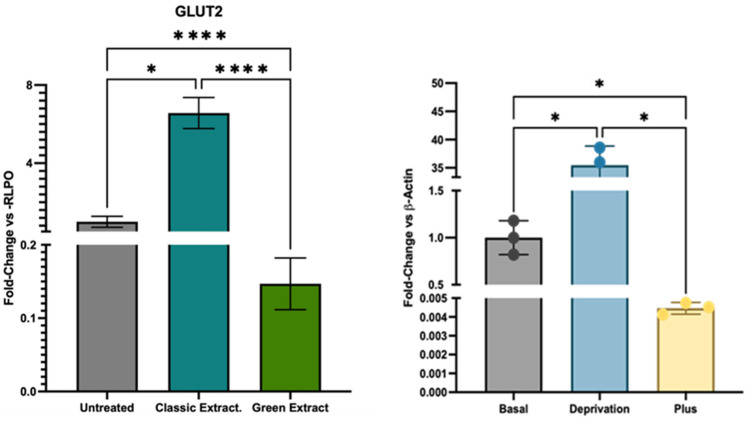
(**Left**) Effects of pistachio-derived extracts on the expression of *GLUT2* (Solute Carrier Family 2) gene in HCT-116 cells after 24 h of treatment. Gene expression levels were assessed by RT-qPCR and normalized to the housekeeping gene RPLP0. Results are expressed as fold change relative to untreated control cells (CTRL). Cells were treated with two types of extracts: the one extracted using a green extraction method, and the one extracted using conventional extraction. (**Right**) The expression of *GLUT2* in HCT-116 cells exposed to three different glucose concentrations: Basal (3 g/L), Deprivation (0 g/L) and Plus (10 g/L) [27]. Statistical significance was determined by Two-stage linear step-up procedure of Benjamini, Krieger and Yekutieli: * *p* < 0.05; ; **** *p* < 0.0001.

Gene expression analysis revealed that treatment with the green extract led to a marked modulation in the expression of the glucose transporter *GLUT2* (Figure 3, Table S2). Specifically, *GLUT2* transcript levels were drastically reduced (fold change: 0.15, *p* < 0.0001) compared to untreated controls, indicating a strong transcriptional downregulation. In contrast, cells treated with the classic extract maintained *GLUT2* expression at levels comparable to control. This sharp decrease in *GLUT2* expression with the green extract aligns with previous findings from our laboratory [27], where HCT-116 cells exposed to excess glucose exhibited significant *GLUT2* downregulation.

The current findings are further substantiated by compositional analysis of the extracts, which revealed a significantly higher carbohydrate content in the green extract (29.6 ± 0.6 mg/g of pistachio material) compared to the classic extract (4.6 ± 0.1 mg/g). This direct chemical evidence supports the hypothesis that the observed reduction in *GLUT2* transcription reflects a cellular adjustment to excess carbohydrate exposure, as a mechanism to limit additional glucose uptake under nutrient-rich conditions.

## 4. Discussion

In recent years, increasing attention has been given to the development of sustainable extraction technologies, with European Green Deal initiatives receiving considerable attention [6]. Nevertheless, it remains essential to assess extraction method selectivity of nutraceutical compounds alongside its environmental and economic benefits.

From a chemical perspective, the differences in yields observed between classical and green extraction methods reflect fundamentally distinct extraction philosophies rather than mere variations in efficiency. UAE employs a polar solvent system recovering a broad spectrum of water-soluble and moderate polar compounds, including sugars and phenolic compounds [35]. In contrast, classical extraction —incorporating additional steps for selective compound isolation [36]—systematically removes co-extracted polar interferents, highlighting the differential selectivity of aqueous and ethanol-based extraction techniques and suggesting enhanced selectivity for phenolic compounds [37].

To investigate the biological consequences of these compositional differences, HCT-116 colon cancer cells were treated with the respective extracts. The green extract induced hyperproliferation after 72 h, leading to rapid confluence and subsequent detachment, likely due to the high sugar content of the extract.

In our study, the results observed upon green extract treatment emphasizes potential risks in pathological contexts, where increased glucose availability may alter cellular metabolism, despite the preservation of anti-inflammatory activity, likely mediated by chlorogenic acid through the upregulation of the anti-inflammatory cytokine *IL-10* and the antioxidant enzyme *HMOX-1* [30,31]. Notably, the polyphenol-rich classical extract induced higher *HMOX-1* expression [38] compared to both the sugar-rich green extract and the control. *HMOX-1*, an inducible enzyme regulated by the Keap1/Nrf2/ARE signaling pathway, responds to oxidative and electrophilic stress [39]. Paradoxically, polyphenols also trigger this endogenous antioxidant response, acting as both free radical scavengers and stimulators of cellular defense mechanisms [40,41]. This dual mode of action strengthens the interpretation that the heightened *HMOX-1* expression induced by the classic extract is driven by polyphenol-mediated activation of the Keap1/Nrf2/ARE pathway [41]. Thus, HCT-116 cells treated with the classical extract exhibited strong activation of the Keap1/Nrf2/ARE pathway, reflecting a robust internal antioxidant response mediated by polyphenols. Importantly, the semi-quantitative profiling of the phenolic fraction provides direct experimental support to this concept, demonstrating that extraction methodology selectively modulates the relative abundance of specific bioactive phenolic subclasses. In particular, the classical extraction protocol resulted in a marked enrichment of oligomeric procyanidins—most notably procyanidin trimers—alongside a relative reduction of chlorogenic acid, whereas the green extraction showed the opposite trend, offering an explanation for the divergent biological responses observed in HCT-116 cells. The increased *HMOX-1* expression observed following treatment with the classical extract is in agreement with previous studies indicating that the antioxidant bioactivity of polyphenols depends, at least in part, on their degree of polymerization. Notably, polyphenol polymerization has been associated with modulation of both direct radical-scavenging activity and the induction of transcriptional antioxidant responses, including activation of the Nrf2/ARE pathway and redox-responsive genes [34,41,42].

Gene expression analysis revealed a pronounced modulation of glucose transport pathways in response to the different pistachio extracts. Notably, cells treated with the green extract exhibited a drastic downregulation of *GLUT2* (fold change: 0.15), a key facilitative glucose transporter involved in maintaining intracellular glucose homeostasis [43]. Such a marked reduction suggests an adaptive cellular response aimed at limiting glucose uptake under conditions of high extracellular carbohydrate availability—consistent with the elevated sugar content identified in the green extract. This finding is particularly relevant in light of previous work from our research group, in which HCT-116 cells subjected to glucose deprivation (0 g/L) markedly overexpressed *GLUT2* [27], enabling the selective internalization of 5-fluorouracil-functionalized glucose based-nanoparticles, while cells exposed to a high-glucose medium (10 g/L) showed a notable reduction in the expression of *GLUT2*, how it has happened with green extract (Figure 3). These data therefore support the notion that *GLUT2* may function as a molecular sensor in HCT-116 cells, being highly plastic and capable of modulating its expression in response to extracellular glucose levels, with potential implications for nutrient sensing and targeted drug delivery strategies.

In parallel, the green extract treatment also led to increased expression of *IL-10*, cytokines associated with anti-inflammatory and immunomodulatory responses. The concurrent downregulation of *GLUT2* and upregulation of this cytokine suggests a coordinated shift in immunometabolic programming, possibly driven by carbohydrate-induced metabolic stress [44,45,46,47], as well as by chlorogenic acid enrichment, that could be linked to different extract selectivity. Thus, the biological effects reported here are not attributable to total phenolic abundance per se, but rather to differences in extract purity and phenolic composition shaped by the extraction strategy.

This pattern was not observed with the classic extract, which maintained *GLUT2* expression at control levels and displayed a distinct gene expression signature dominated by *HMOX-1* induction.

In summary, while increased carbohydrate content might be beneficial in non-pathological contexts (for example, providing readily available energy to help athletes maintain performance and redox balance [48,49,50]), the intended therapeutic or nutraceutical applications of pistachio extracts should be carefully considered in metabolic disorders or cancer patients. In these contexts, where cancer cells exhibit constitutively elevated glycolytic fluxes (Warburg effect) [51], excess of readily metabolizable substrates could unintentionally support tumor proliferation. However, co-extracted non-target metabolites are not inherently detrimental; nonetheless, by augmenting metabolic fluxes and facilitating substrate uptake, they can promote a high-energy cellular state. Such a condition may be advantageous in physiological settings but could prove counterproductive in oncological contexts, where additional metabolic stimulation may potentiate tumor cell growth and survival rather than yield therapeutic benefit [9,52,53,54].

While green extraction methods are certainly in line with sustainability goals—owing to their enhanced extraction yields compared with classic techniques and the reduced reliance on toxic organic solvents—their potential to extract unwanted components requires further optimization [55,56]. A careful refinement of green extraction methods [53] is crucial to achieve a balance between environmental sustainability, purity of extracts and therapeutic efficacy. These findings underscore an important consideration for nutraceutical development: while green extraction technologies offer environmental advantages, the selectivity of the extraction process critically influences the biochemical profile of the final product, which in turn determines its biological activity and therapeutic applicability.

## 5. Conclusions

This study highlights the significant impact of extraction methodology on the chemical composition and biological activity of pistachio-derived extracts. Although green extraction methods are environmentally advantageous, this study revealed that they lead to higher carbohydrate content, which induces hyperproliferative responses in HCT-116 colon cancer cells, despite the preservation of anti-inflammatory activity. These effects were evidenced by increased cell confluence and detachment, downregulation of *GLUT2*, and upregulation of anti-inflammatory cytokine such as *IL-10* and *HMOX-1*. In contrast, extracts obtained through classical methods demonstrated a strong antioxidant profile, as reflected by enhanced *HMOX-1* expression, without exerting any detectable effect on cell proliferation. Collectively, these findings highlight the need for precise control over extraction parameters to ensure the safety and efficacy of pistachio-based nutraceuticals, particularly in pathological contexts such as cancer. In future applications, the therapeutic potential of these extracts in in vivo models should be explored so that their biological activity can be validated, and systemic responses can be assessed. Additionally, industrial applications could benefit from optimized green extraction processes that preserve bioactivity while aligning with the sustainability goals of the Green Deal.

Despite the promising results, this study has several limitations. First, the exclusive use of a single colorectal cancer cell line may limit the generalizability of the findings to other tumor models. Second, comprehensive metabolomic analyses are still required to accurately characterize the molecular pathways influenced by the extract composition and to identify the specific bioactive compounds responsible for the observed effects. Third, the lack of in vivo validation precludes conclusions regarding systemic efficacy, bioavailability, and safety. These considerations underscore the importance of conducting future translational studies to assess the clinical relevance and therapeutic potential of pistachio-derived extracts across a broader range of biological systems. In this context, ongoing experiments will also include expanded cytokine profiling to better define the immunomodulatory activity of the different extracts.

## Figures and Tables

**Table 1:** Primers used for RT-qPCR in this study. All primers were ordered from GeneGlobe, QIAGEN (cat. No. 249990).

Official Name	Official Symbol	Alternative Titles/Symbols	Detected Transcript	Amplicon Lenght
Ribosomal Protein Lateral Stalk Subunit P0	RPLP0	PRLP0; P0; L10E; RPP0; LP0	NM_053275 NM_001002	68 bp
Heme Oxygenase 1	*HMOX-1*	HO-1	NM_002133	161 bp
Solute Carrier Family 2 Member 2	*GLUT2*	*GLUT2*; Solute Carrier Family 2 Member 2; Glucose Transporter Type 2, Liver	NM_000340; NM_001278658; NM_001278659	120 bp
Interleukin 6	*IL-6*	*IL-6*	NM_031168	128 bp
Interleukin 10	*IL-10*	*IL-10*	NM_000572	112 bp

**Table 2:** Semi-quantitative polyphenolic composition of Bronte pistachio extracts, expressed as % relative abundance calculated from peak area ratios (mean values from triplicate injections).

Compound (RT, min)	UAE (Green)	Classic
Luteolin derivative (45.5)	16.7%	17.2%
Rutin (58.3)	61.1%	62.9%
Chlorogenic acid (61.3)	16.2%	5.8%
Procyanidin trimer (67.3)	6.1%	14.1%

**Table 3:** Carbohydrate content in pistachio extracts. * Expressed as glucose equivalents per 100 mg dry extract (mean ± SD, *n* = 3); ** Calculated as (% carbohydrates in extract × extraction yield)/100.

Extract Origin	Extraction Method	Extraction Yield (%)	Carbohydrates in Extract (% *w*/*w*) *	Carbohydrates per g Pistachio (mg/g) **
Bronte	UAE (Green)	8.8	33.6 ± 0.8	29.6 ± 0.6
	Classic	1.8	25.5 ± 0.6	4.6 ± 0.1

## Data Availability

The original contributions presented in this study are included in the article/Supplementary Materials. Further inquiries can be directed to the corresponding author.

## Supplementary Materials

The following supporting information can be downloaded at: https://www.mdpi.com/article/10.3390/foods15020205/s1, TableS1, Details of the ordinary one-way ANOVA multiple comparisons test performed on the MTT assay results at 24, 48, and 72 hours; TableS2, Results of the two-stage linear step-up procedure of Benjamini, Krieger, and Yekutieli for multiple comparisons of IL-6, IL-10, HMOX1, and GLUT2 expression levels; Table S3, RNA Integrity Number (RIN) analysis results performed after RNA extraction; Figure S1, HPLC-MS chromatogram of pistachio green extract. Peak assignments: 45.5 min (luteolin derivative), 58.3 min (rutin), 61.3 min (chlorogenic acid), 67.3 min (procyanidin trimer); Figure S2, HPLC-MS chromatogram of pistachio classic extract. Peak assignments: 45.5 min (luteolin derivative), 58.3 min (rutin), 61.3 min (chlorogenic acid), 67.3 min (procyanidin trimer).

## Abbreviations

The following abbreviations are used in this manuscript:

UAE	Ultrasound-Assisted Extraction
GC	Gas Chromatography
HPLC	High-Performance Liquid Chromatography
qPCR	Quantitative Polymerase Chain Reaction
RNA	Ribonucleic Acid
DNA	Deoxyribonucleic Acid
*IL-6*	Interleukin 6
*IL-10*	Interleukin 10
*HMOX-1*	Heme Oxygenase 1
*GLUT2*	Glucose Transporter Type 2
ROS	Reactive Oxygen Species
DMEM	Dulbecco’s Modified Eagle Medium
FBS	Fetal Bovine Serum
PBS	Phosphate-Buffered Saline
MTT	3-(4,5-Dimethylthiazol-2-yl)-2,5-diphenyltetrazolium bromide
SD	Standard Deviation
ANOVA	Analysis of Variance
CV	Coefficient of Variation
