# Supplementary material for: Influence of Extraction Method on the Bioactivity of Pistacia vera L. Extracts: Metabolic and Gene Expression Changes in Colorectal Cancer Cells
Source: Foods. 2026 Jan 7;15(2):205. doi: 10.3390/foods15020205 (PMC12840026; doi:10.3390/foods15020205)

SUPPLEMENTARY MATERIALS

Influence of Extraction Method on the Bioactivity of Pistachio Extracts: Metabolic and Gene Expression Changes in Colorectal Cancer Cells

Giulia Zerbo[a], Paolo Giuseppe Bonacci[b,d]*, Morena Terrana[b], Valentina Greco[a], Gianfranco Cavallaro[a], Carmela Bonaccorso[a], Cosimo Gianluca Fortuna[a], Nicolò Musso[c,d].

Table S1. Details of the ordinary one-way ANOVA multiple comparisons test performed on the MTT assay results at 24, 48, and 72 hours.

24 hours								
Dunnett's multiple comparisons test	Mean Diff,	95,00% CI of diff,	Significant?	Summary	Adjusted P Value	A-?		
Untreated vs. Green Extract	-4,839	-11,62 to 1,948	No	ns	0,1812	B	Green Extract	
Untreated vs. Classic Extract	-9,657	-17,97 to -1,346	Yes	*	0,0226	C	Classic Extract	
Test details	Mean 1	Mean 2	Mean Diff,	SE of diff,	n1	n2	q	DF
Untreated vs. Green Extract	100,0	104,8	-4,839	2,801	4	4	1,727	17
Untreated vs. Classic Extract	100,0	109,7	-9,657	3,431	4	4	2,815	17

48 hours								
Dunnett's multiple comparisons test	Mean Diff,	95,00% CI of diff,	Significant?	Summary	Adjusted P Value	A-?		
Untreated vs. Green Extract	9,940	5,964 to 13,92	Yes	****	<0,0001	B	Green Extract	
Untreated vs. Classic Extract	17,87	13,00 to 22,74	Yes	****	<0,0001	C	Classic Extract	

Test details	Mean 1	Mean 2	Mean Diff,	SE of diff,	n1	n2	q	DF
Untreated vs. Green Extract	100,0	90,06	9,940	1,641	4	4	6,057	17
Untreated vs. Classic Extract	100,0	82,13	17,87	2,010	4	4	8,890	17

72 hours								
Dunnett's multiple comparisons test	Mean Diff,	95,00% CI of diff,	Significant?	Summary	Adjusted P Value	A-?		
Untreated vs. Green Extract	37,08	22,16 to 52,00	Yes	****	<0,0001	B	Green Extract	
Untreated vs. Classic Extract	-4,697	-22,97 to 13,57	No	ns	0,7703	C	Classic Extract	

Test details	Mean 1	Mean 2	Mean Diff,	SE of diff,	n1	n2	q	DF
Untreated vs. Green Extract	100,0	62,92	37,08	6,158	4	4	6,022	17
Untreated vs. Classic Extract	100,0	104,7	-4,697	7,542	4	4	0,6228	17

Table S2. Results of the two-stage linear step-up procedure of Benjamini, Krieger, and Yekutieli for multiple comparisons of IL-6, IL-10, HMOX1, and GLUT2 expression levels.

IL6					
Two-stage linear step-up procedure of Benjamini, Krieger and Yekutieli	n	Mean diff,	Discovery?	q value	Individual P Value
Untreated vs. Green Extract	3	-0,2682	No	0,1203	0,3436
Untreated vs. Classic Extract.	3	0,8024	Yes	0,0088	0,0168
Untreated vs. PB4	3	-1,026	Yes	0,0051	0,0049
IL-10					
Two-stage linear step-up procedure of Benjamini, Krieger and Yekutieli	n	Mean diff,	Discovery?	q value	Individual P Value
Untreated vs. Green Extract	3	-4,456	Yes	<0,0001	<0,0001
Untreated vs. Classic Extract.	3	0,7262	No	0,1337	0,191
Untreated vs. PB4	3	-1,026	No	0,0821	0,0782
HMOX-1					
Two-stage linear step-up procedure of Benjamini, Krieger and Yekutieli	n	Mean diff,	Discovery?	q value	Individual P Value
Untreated vs. Green Extract	3	-1,842	Yes	0,002	0,0039
Untreated vs. Classic Extract.	3	-3,792	Yes	<0,0001	<0,0001
Untreated vs. PB4	3	-1,026	Yes	0,0195	0,0556
GLUT2					

Two-stage linear step-up procedure of Benjamini, Krieger and Yekutieli	n	Mean diff,	Discovery?	q value	Individual P Value
Untreated vs. Green Extract	3	0,8531	Yes	0,0263	0,0751
Untreated vs. Classic Extract.	3	-5,568	Yes	<0,0001	<0,0001
Green Extract vs. Classic Extract.	3	-6,421	Yes	<0,0001	<0,0001

Table S3. RNA Integrity Number (RIN) analysis results performed after RNA extraction.

Sample	RNA Integrity Number	Quantification (ng/µL)
HCT-116 Control	9.1	184
HCT-116 treated with Green Extract	9.5	284
HCT-116 treated with Classic Extract	10	177

Figure S1. HPLC-MS chromatogram of pistachio green extract. Peak assignments: 45.5 min (luteolin derivative), 58.3 min (rutin), 61.3 min (chlorogenic acid), 67.3 min (procyanidin trimer).

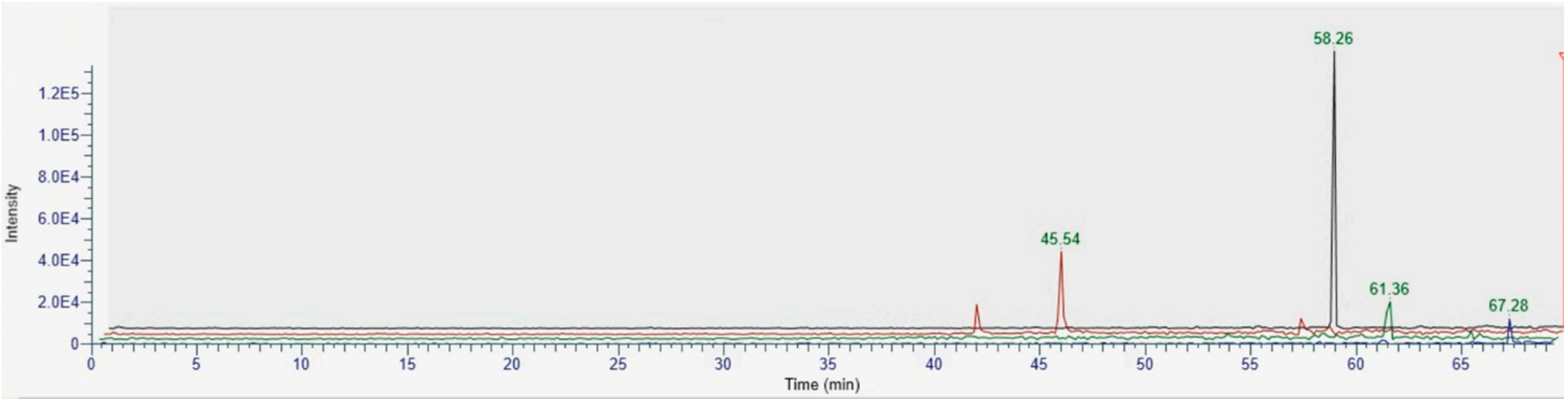

Figure S2. HPLC-MS chromatogram of pistachio classic extract. Peak assignments: 45.5 min (luteolin derivative), 58.3 min (rutin), 61.3 min (chlorogenic acid), 67.3 min (procyanidin trimer).

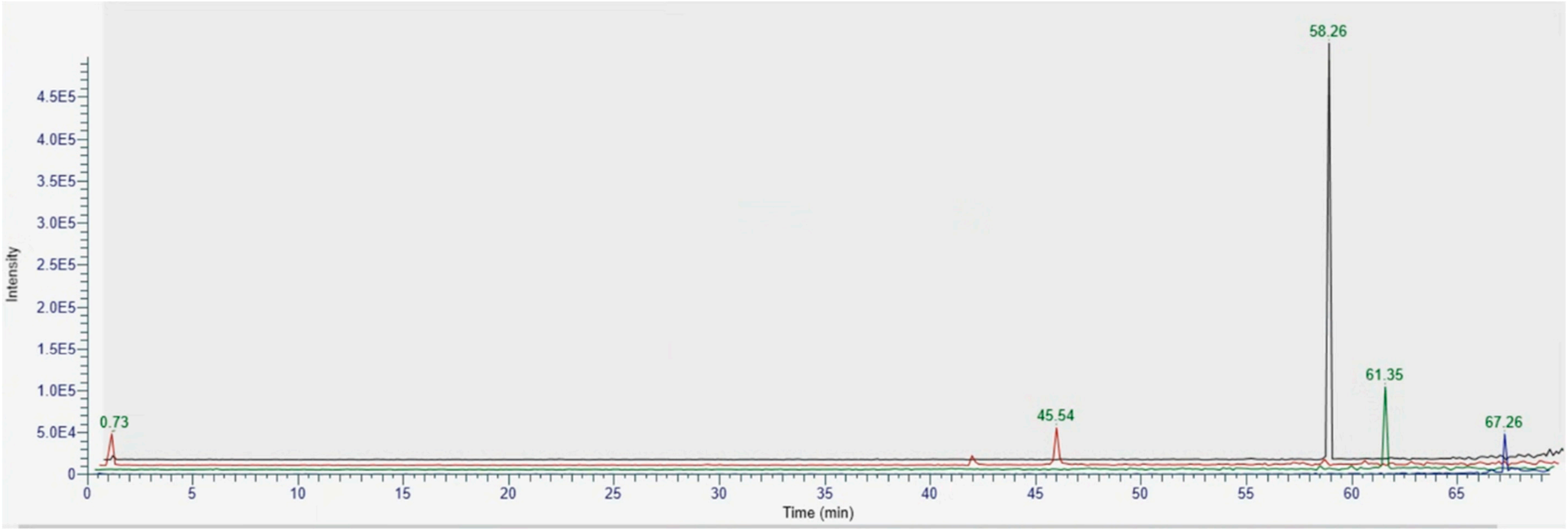

Supplement: Supplementary file 1 [file foods-15-00205-s001.zip › foods-3989997-supplementary.pdf]
